# New Taxonomy and the Origin of Species

**DOI:** 10.1371/journal.pbio.0050194

**Published:** 2007-07-17

**Authors:** Shai Meiri, Georgina M Mace

## Abstract

The trend for elevating known subspecies to the status of species on the basis of inappropriate evidence can potentially divert important conservation funds away from other species.

On March 15th 2007, the World Wildlife Fund announced a new species of clouded leopard, Neofelis diardi, from Borneo and Sumatra. The media was enthusiastic; the Times of London, for example, published a picture of the new species on its front page, declaring this to be the first new species of big cat to be identified “in almost two centuries.” Disappointingly however, N. diardi is far from new. It was described by Cuvier in 1823, then relegated to a subspecies of the mainland species N. nebulosa. Recent morphological [1] and genetic [2] studies, however, now suggest that it is sufficiently distinct to merit specific status.

Describing new species of mammals is an increasingly common event; a process sometimes referred to as “taxonomic inflation” [3–5]. The total number of mammal species has risen from 4,659 in 1993 to 5,418 in 2005 [6,7] and the announcement of Neofelis diardi exemplifies the trend for increased species recognition, based not on new discoveries in the wild but on the elevation of known allopatric subspecies (i.e., with no geographic overlap) to species. While we welcome the stronger support for conservation that species status will provide, it is important that species status be assigned appropriately.

Most of these recently described species are allopatric or parapatric (i.e., with ranges that abut but do not overlap) populations, separated by barriers such as rivers. Given a barrier to gene flow, the accumulation of genetic and morphological differences is expected [8] and may be of limited biological importance. It seems, however, that many recent taxonomic studies regard the presence of allopatric populations as an indication that speciation has occurred. We suggest that stronger evidence is needed to show that populations are sufficiently distinct to merit specific status. This evidence should be capable of discriminating genuine ecological and evolutionary distinctiveness from minor differences that could result from geographic isolation [9]. Mayr [8] claimed that taxonomy has three major impacts on evolutionary thought: Advancing the predominance of allopatric speciation, introducing the biological species concept, and indicating the prevalence of polytypic species (Rassenkreis [8,10]) that vary over geographic space. The current trend of splitting species endorses the first, ignores or disagrees with the second, and denies the third.

In resurrecting N. diardi, Kitchener et al. [1] are relying on the phylogenetic species concept whereby species are defined as groups that share at least one uniquely derived character. They distinguish two clouded leopard “species” solely on the basis of pelage (fur color and pattern) characteristics, despite the fact that differences in hair color often reflect minor geographical varieties in many mammals. Borneo and the Malay Peninsula differ in several biotic and abiotic factors. Thus genetic and morphological differences between populations of the 144 mammalian species they share [11] are to be expected, and there could potentially be equivalent evidence to merit specific status for all of these; an outcome that would certainly be unjustified.

The biological species concept is broadly inapplicable for allopatric populations separated by barriers. Regarding any derived character as conferring specific status is, however, not justifiable. Using such criteria, we would see a return to the taxonomic practices of the era of Merriam [12], who split North American brown bears into 82 species (in two genera), promoting GG Simpson to remark that Merriam “had a (fortunately) unique conception of the character of a species, giving it less scope than most authors give a minor geographic race, not much more than an individual genetic family group. On such a system twin bear cubs could be of different species” [13]. North American brown bears are nowadays believed to represent two subspecies in a single Holarctic species, Ursus arctos, but if any derived character is enough to confer species status, then certainly Merriam was closer to the truth.

We suggest re-introducing the notion of the polytypic species; putting the Rassenkreis into taxonomy. Genetic and morphological differences between populations are expected to evolve in allopatry but should be substantial to merit specific status. Although there are no a priori criteria for just how different populations have to be to be called species, geographic variation should certainly be taken into consideration.

Splitting allopatric populations into species makes each of these more vulnerable than the polytypic species has been, because ranges and population sizes are smaller [3]. If, as seems likely from the World Wildlife Fund announcement, conservation resources are directed towards newly identified endangered species, then accepting N. diardi will benefit the conservation of Bornean and Sumatran clouded leopards. Conservation funds are however limited, so this may in fact be achieved by diverting funds away from other species.. We are not suggesting that there should necessarily be reduced conservation for these forms. Conservation actions should support species across their ranges, perhaps favoring phenotypically distinct populations or geographically isolated subsets so as to fully conserve variation. Rather, we note that splitting species per se does not necessarily have conservation value [14].

Where should the burden of proof lie when naming new species? Given the importance of species designation for conservation and for comparative studies that contribute to our understanding of biodiversity, we believe species status needs to be awarded after careful consideration of the evidence to support its biological significance based on morphological, geographical, ecological, behavioral, and genetic information [15]. Furthermore, the choice of characters used in classification should not be focused on highly labile traits that show clear patterns of geographic variation [16]. Simply identifying differences is not enough; a quantitative comparative approach should show (as in [2]) that the degree of observed differences is similar to differences observed between closely related sympatric (i.e., geographically overlapping) species.

We should celebrate the discovery of new species when they genuinely add to the pool of evolutionary diversity (e.g., [17]), but we must be careful not to simply reduce the threshold. In practice, we suggest that when splitting previously recognized polytypic species, taxonomists present sufficient evidence that morphological, ecological, behavioral, and genetic differences between the two forms are of a magnitude that would merit specific rank in closely related sympatric forms.
